# Gradient Spin Echo (GraSE) imaging for fast myocardial T2 mapping

**DOI:** 10.1186/s12968-015-0127-z

**Published:** 2015-02-12

**Authors:** Alois M Sprinkart, Julian A Luetkens, Frank Träber, Jonas Doerner, Jürgen Gieseke, Bernhard Schnackenburg, Georg Schmitz, Daniel Thomas, Rami Homsi, Wolfgang Block, Hans Schild, Claas P Naehle

**Affiliations:** Department of Radiology, University of Bonn, Sigmund-Freud-Straße 25, 53127 Bonn, Germany; Institute of Medical Engineering, Ruhr-University Bochum, Universitätsstraße, Bochum, Germany; Philips Healthcare Germany, Lübeckertordamm, Hamburg, Germany

**Keywords:** Cardiovascular magnetic resonance, T2 mapping, Phantom study, Quantitative MRI, Gradient-spin-echo imaging

## Abstract

**Background:**

Quantitative Cardiovascular Magnetic Resonance (CMR) techniques have gained high interest in CMR research. Myocardial T2 mapping is thought to be helpful in diagnosis of acute myocardial conditions associated with myocardial edema. In this study we aimed to establish a technique for myocardial T2 mapping based on gradient-spin-echo (GraSE) imaging.

**Methods:**

The local ethics committee approved this prospective study. Written informed consent was obtained from all subjects prior to CMR. A modified GraSE sequence allowing for myocardial T2 mapping in a single breath-hold per slice using ECG-triggered acquisition of a black blood multi-echo series was developed at 1.5 Tesla. Myocardial T2 relaxation time (T2-RT) was determined by maximum likelihood estimation from magnitude phased-array multi-echo data. Four GraSE sequence variants with varying number of acquired echoes and resolution were evaluated in-vitro and in 20 healthy volunteers. Inter-study reproducibility was assessed in a subset of five volunteers. The sequence with the best overall performance was further evaluated by assessment of intra- and inter-observer agreement in all volunteers, and then implemented into the clinical CMR protocol of five patients with acute myocardial injury (myocarditis, takotsubo cardiomyopathy and myocardial infarction).

**Results:**

In-vitro studies revealed the need for well defined sequence settings to obtain accurate T2-RT measurements with GraSE. An optimized 6-echo GraSE sequence yielded an excellent agreement with the gold standard Carr-Purcell-Meiboom-Gill sequence. Global myocardial T2 relaxation times in healthy volunteers was 52.2 ± 2.0 ms (mean ± standard deviation). Mean difference between repeated examinations (n = 5) was −0.02 ms with 95% limits of agreement (LoA) of [−4.7; 4.7] ms. Intra-reader and inter-reader agreement was excellent with mean differences of −0.1 ms, 95% LoA = [−1.3; 1.2] ms and 0.1 ms, 95% LoA = [−1.5; 1.6] ms, respectively (n = 20). In patients with acute myocardial injury global myocardial T2-RTs were prolonged (mean: 61.3 ± 6.7 ms).

**Conclusion:**

Using an optimized GraSE sequence CMR allows for robust, reliable, fast myocardial T2 mapping and quantitative tissue characterization. Clinically, the GraSE-based T2-mapping has the potential to complement qualitative CMR in patients with acute myocardial injuries.

**Electronic supplementary material:**

The online version of this article (doi:10.1186/s12968-015-0127-z) contains supplementary material, which is available to authorized users.

## Background

Cardiovascular Magnetic Resonance (CMR) can provide safe and prompt diagnosis in patients with acute myocardial injury (e.g. acute myocardial infarction, tako tsubo cardiomyopathy and acute myocarditis) [1,2]. Many acute myocardial conditions lead to myocardial edema that increases the free myocardial water content. Myocardial water content is directly related to myocardial T2 relaxation times [3], thus it can be visualized using black blood T2-weighted (T2-w) imaging [4]. However, this qualitative technique is challenging and has several limitations that compromise its use in clinical routine [5]: (1) The black blood impulse can result in an imperfect nulling of the left ventricular blood pool signal making it difficult to differentiate subendocardial edema from low flow left ventricular blood; (2) Arrhythmia and the use of phased array coils can cause signal intensity inhomogeneities that may obscure myocardial edema; (3) In cases of diffuse global myocardial edema, when no signal from healthy myocardium is present, correct image interpretation may also be hampered.

Quantitative T2 mapping techniques may overcome some of these limitations in qualitative T2-w imaging. During the last decades, several techniques and sequences for myocardial T2 mapping have been described [6-9] with several studies defining normal values for healthy human myocardium [7,9-13]. However, some of these methods are either time-consuming, are acquired in free breathing, or require specialized software for data acquisition and/or post-processing, all of which are factors that limits their use in clinical routine. Measurement of an absolute tissue property for the quantification of myocardial edema is not only expected to be beneficial for establishing the diagnosis but can also improve monitoring and help in guiding therapy, if well defined T2 mapping sequences are available, that allow for good quality, reliable and rapid data acquisition.

In summary, a readily available, phantom-validated T2 mapping sequence with high accuracy and reproducibility in healthy volunteers is desirable allowing for a good discrimination between diseased and healthy myocardium. Therefore, the purpose of this study was to develop and evaluate a sequence for accurate myocardial T2 mapping using the gradient-spin-echo (GraSE) technique [14,15] which (1) has an acceptable acquisition time for integration into clinical CMR protocols, (2) may overcome some of the limitations of standard T2-w imaging, and (3) utilizes standard imaging and post-processing methods that allow a widespread clinical implementation.

## Methods

The local ethic committee approved this prospective study and written informed consent was obtained from all study subjects prior to CMR. All scans were performed using a 1.5 Tesla (T) MR system (Ingenia 1.5 T, Philips Healthcare, Best, The Netherlands) with a maximum gradient strength of 45 mT/m and a maximum slew rate of 120 mT/m/ms. A 32 channel torso coil with digital interface was used for signal reception.

### T2 Mapping sequence details

For pixel-wise measurement of T2 relaxation times, a multi-echo dataset was acquired based on two well established MR techniques: (1) Turbo-spin-echo (TSE), originally dubbed rapid acquisition with relaxation enhancement (RARE) [16] and also known as fast-spin-echo, and (2) echo-planar-imaging (EPI) [17]. In this sequence a train of spin-echoes is generated by several 180° radiofrequency pulses and each individual spin echo is acquired with an EPI readout. This concept is known as gradient-spin-echo. In contrast to the conventional GraSE sequence, that can be utilized to further speed up TSE sequences and/or to reduce energy deposition, here each of the sampled echoes in the TSE echo-train is used for the reconstruction of a separate image with varying effective echo time (TE). Consequently, the number of 180° pulses (TSE factor) determines the number of images in the multi-echo series, and the EPI factor (specifying the number of gradient echoes per readout) determines the number of profiles acquired per spin-echo. An illustration of the sequence can be found in the Additional file 1.

A standard dual inversion recovery black blood module was applied to null the signal of flowing blood in the ventricles; the inversion delay was calculated automatically from the cardiac frequency assuming a T1 relaxation time of 1200 ms for blood at 1.5 T. The bandwidth of the second inversion pulse was increased to achieve complete re-inversion in the myocardium (with a slice thickness for re-inversion of 2.5 fold the imaging slice thickness). To avoid ghosting artifacts and possible signal contributions from pericardial or subcutaneous fat, which can severely confound T2 measurements, fat suppression was applied using a spectral selective inversion recovery pulse (SPIR).

Signal acquisition was triggered by ECG and data were acquired during breath-hold. With parallel imaging the acquisition time was reduced to a maximum of 13 RR intervals per slice including one dummy TR. Hence, data could be acquired in a single breath-hold per slice even in cases with a low cardiac frequency. Finally, T2 maps were generated directly on the scanner applying a maximum likelihood estimation on the magnitude phased-array multi-echo data taking the non-Gaussian distribution of noise in magnitude images into account [18,19].

### Phantom experiments

To test the accuracy of the method, six phantom tubes were filled with Manganese (II) chloride doped water (MnCl_2_ · 4 H_2_0) in various concentrations covering the whole range of T2 relaxation times relevant for myocardial T2 mapping. With a T1 relaxation time of about 10 times T2 at 1.5 T, MnCl_2_ · 4 H_2_0 solution is a well-suited phantom for sequence optimization.

A phantom pre-study was performed to determine a suitable set of sequence parameters for the GraSE multi-echo sequence (see Additional file 1). T2 values obtained from the measurements with the GraSE sequence were compared to the T2 values determined by a Carr-Purcell-Meiboom-Gill sequence (see Additional file 1), which was considered as the Gold standard.

### In-vivo studies

Based on the phantom experiments, four sets of sequence parameters were selected for in-vivo evaluation in volunteers: a nine echo variant (9 Ec), a six-echo variant (6 Ec), both with one start-up echo (i.e. the first echo is not used for data acquisition), a 6 echo variant with lower resolution (6 Ec LR) and therefore higher signal to noise ratio (SNR), and a 7 echo variant with the same setting as the 6 Ec variant but without a start-up echo (7 Ec no s.e.). Sequence details are listed in Table 1. Accounting for the results of the phantom experiments, modified Shinnar-Le-Roux pulses for optimized slice refocusing with a sinc-gauß like shape and modified side-lobes (one at each side) were applied with a duration of 4.5 ms.

**Table 1:** **Details of the GraSE sequences**

	9 Ec	**6 Ec** (LR)	7 Ec no s.e.
Acquisition matrix	**176 × 168** (152 × 138)		
Voxel size	**2 × 2 mm** ^**2**^ (2.3^2^) **reconstr. to 1 × 1 mm** ^**2**^		
Slice thickness	**10 mm**		
TR	**1 RR interval**		
First TE	23.6 ms	**23.6 ms**	11.8 ms
∆ TE	**11.8 ms**		
Number of echoes	9	**6**	7
EPI/SENSE factor	**7/2**		
Black blood/Fat sat	**Dual Inversion/SPIR**		
Profile order	**Linear**		
TSE shot duration	118 ms	**83 ms**	83 ms
Time shift between first and last echo	94.4 ms	**59 ms**	70.8 ms
Bandwidth (P/F)	**173 Hz/1740 Hz**		

Values in bold correspond to the six-echo variant proposed for clinical application and which was analyzed in detail.

T2 mapping with all four sequence variants was performed in 20 healthy volunteers. All healthy volunteers had no medical history of cardiac or vascular disease and no cardiac risk factors. ECG-analyses showed no abnormalities in all volunteers. Breath-hold T2 mapping was performed in end-diastole in short axis orientation. Three short axis slices (basal, mid-ventricular, and apical) were obtained for coverage of all 16 segments as described previously [20].

Myocardial T2 relaxation times were extracted from the T2 maps by using freely available software (Segment, version 1.9, R2783; http://segment.heiberg.se) [21]. After endocardial and epicardial borders were contoured by two readers with 2 (JAL) and 9 (CPN) years of experience in CMR, T2 maps were analyzed by a segmental approach according to the 16 segment AHA model [20].

To assess the inter-study repeatability of the different GraSE sequence variants, myocardial T2 mapping was repeated in five randomly chosen volunteers within a two week period. Based on the results of the phantom and the in-vivo study, the performance of the six-echo GraSE sequence was further evaluated by an assessment of the intra- as well as inter-reader agreement in all volunteers.

For functional analysis ECG-gated steady-state free precession short axis cine images covering the whole left ventricle were obtained in breath-hold. Left ventricular end systolic volume and left ventricular ejection fraction were quantified by using dedicated software (ViewForum, Philips Healthcare). Papillary muscles were included in the left ventricular cavity volume.

### Clinical application

In order to demonstrate potential clinical applications for myocardial T2 mapping with GraSE, the six-echo T2 mapping sequence was implemented into the CMR protocol of 5 patients with acute myocardial injury. Two patients with acute myocardial infarction, two patients with acute myocardial inflammation, and one patient with takotsubo cardiomyopathy were examined. CMR protocol included black blood T2-w short tau inversion-recovery (STIR) sequences for detection of myocardial edema and segmented inversion-recovery gradient-echo sequences for detection of myocardial fibrosis and scarring. The diagnoses of acute myocardial infarction, acute myocarditis, and takotsubo cardiomyopathy were made on the basis of the results of CMR, electrocardiogram, coronary angiography and serum markers indicating acute myocardial injury and inflammation (troponin and C-reactive protein, respectively).

### Image quality analysis

Image quality (under special consideration of motion artifacts) of the six-echo T2 mapping variant was assessed in the given study population (20 healthy volunteers and 5 patients with acute myocardial conditions). One reader (9 years of experience in CMR) blinded to the patient/volunteer information rated the T2 maps (basal, midventricular, and apical sections) using the following 5-point rating scale: 1: non-diagnostic, 2: poor, severe motion artifacts and/or misregistration compromising image quality and severely hampering analysis, 3: moderate, with minor motions artifacts and/or misregistration at the myocardial borders with moderate effect on image analysis, 4: good, minimal motion or misregistration artifacts with minimal effect on image analysis, 5: excellent, no artifacts.

### Statistical analysis

Statistical analysis was performed using SPSS V22 (IBM Corp, Armonk, NY). Continuous variables are presented as mean ± standard deviation (SD), dichotomous variables are presented as absolute frequency. The paired and unpaired Student’s t-Test was used for comparison of continuous variables. The level of statistical significance was set to p < 0.05. Bonferroni correction was used as an adjustment for multiple comparisons. Bland-Altman analysis and Pearson correlation were performed to assess the agreement of measurements.

## Results

### Phantom experiments

**Figure 1:**
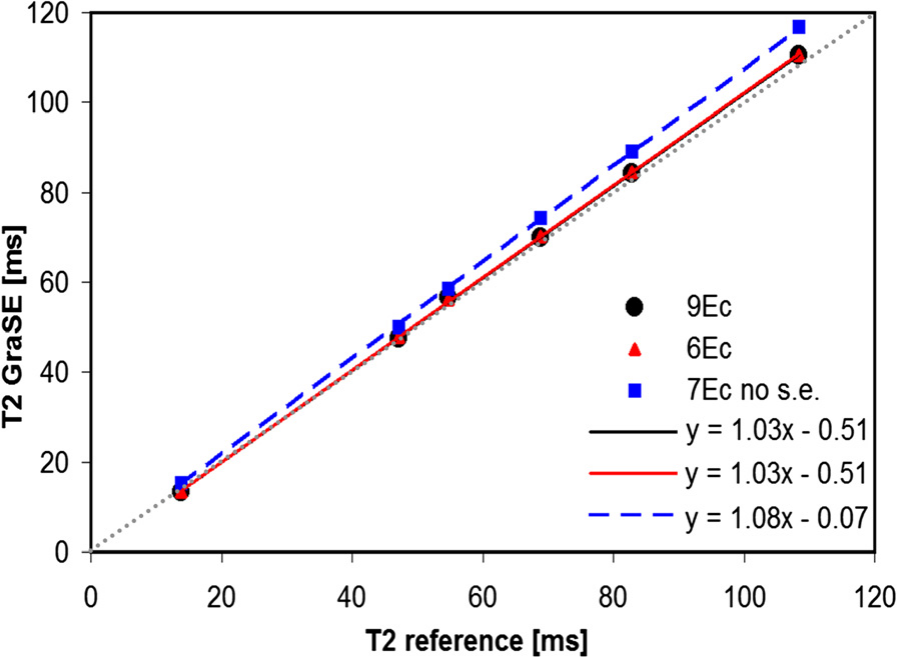
**Results of phantom measurements.** The agreement between T2 measurements using the GraSE sequence and the reference (CPMG sequence) were excellent for the 9 Echo and the 6 Echo variant with the parameter “slice profile” set to “optimal” and one start-up echo. Without start-up echo, T2 relaxation times were slightly overestimated (7Ec no s.e.). The dotted line in gray represents the line of identity corresponding to a perfect agreement with the reference.

The phantom study demonstrated the need for well defined sequence settings for T2 mapping with GraSE to obtain accurate measurements. Details of the phantom measurements can be found in the Additional file 1. With optimized parameters, i.e. especially with appropriate slice profile settings, T2 values obtained by T2 mapping with GraSE showed an excellent correlation with the Carr-Purcell-Meiboom-Gill sequence for each of the four sequence variants (R >0.999, each). The sequence variant without start-up echo led to a slight overestimation of the T2 relaxation times while the nine- and six-echo variant showed almost perfect agreement with the reference (see Figure 1).

### In-vivo studies

**Figure 2:**
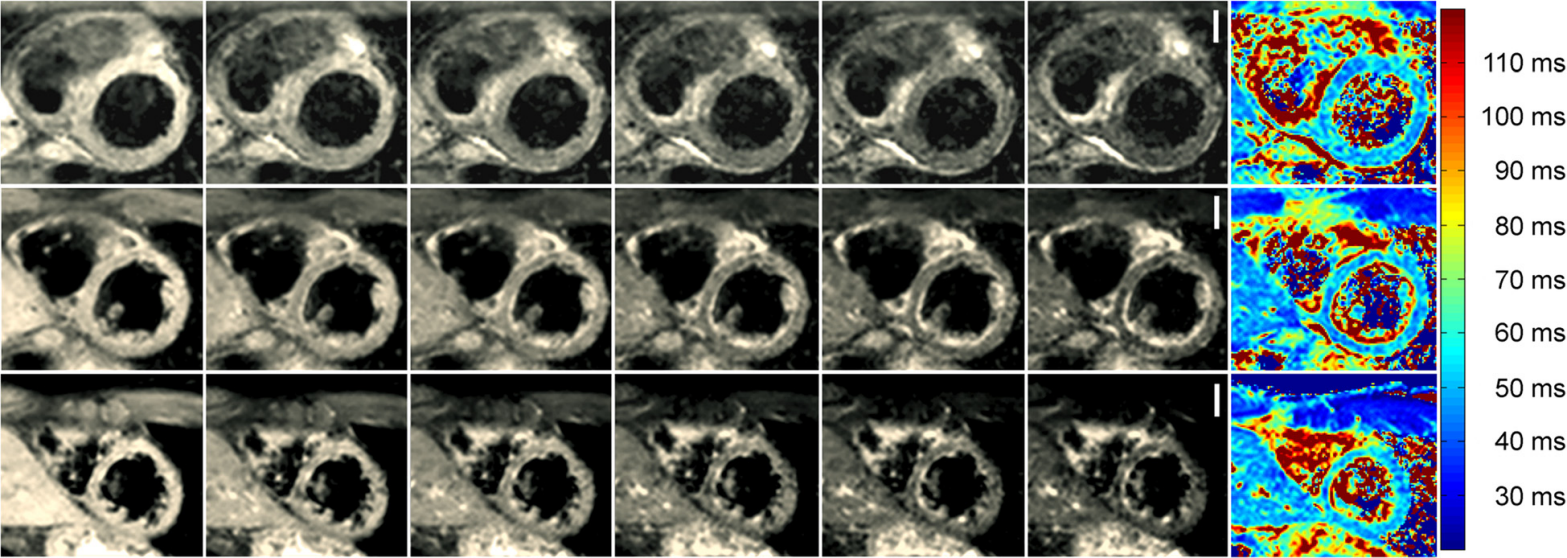
**Results of the six-echo GraSE sequence with corresponding T2 maps for a healthy 37-year-old male.** Each row shows a single slice (from top to bottom: basal, mid-ventricular, apical slice) with echo-times ranging from 23.6 ms to 82.6 ms (from left to right). Data were acquired in one breath-hold per slice (12.8 s). No motion correction was applied. Color-coded T2 maps are shown in the last column and were generated based on a maximum likelihood estimation accounting for the non-Gaussian distribution of noise in magnitude images. Global myocardial T2 relaxation time in this subject was 51.6 ± 3.3 ms (basal anterior/anteroseptal/inferoseptal/inferior/inferolateral/anterolateral: 50.1/51.6/47.6/47.7/45.0/52.9 ms; midventricular anterior/anteroseptal/inferoseptal/inferior/inferolateral/anterolateral: 55.5/57.1/50.2/50.2/54.6/49.2 ms; apical anterior/septal/inferior/lateral: 54.7/54.8/49.4/54.9 ms). To ease comparison with clinical cases (see Figure 5), color-settings are kept equal throughout the article. Scale bars equal 20 mm.

Myocardial T2 mapping with four variants of the GraSE sequence was performed in 20 healthy volunteers (17/20 [85%] men and 3/20 [15%] women). Mean age was 32.4 ± 8.1 years (range: 18 to 50 years). Mean left ventricular ejection fraction was 62.1 ± 4.0% (left ventricular end diastolic volume/body surface area: 76.9 ± 8.1 ml/m^2^; heart rate: 64.7 ± 7.2 beats/minute). Mean breath-hold duration was 12.2 s with a maximum of 14.8 s. All myocardial segments were included into segmental analysis (1280/1280 [100%]). No segments were excluded from analysis. Figure 2 shows a GraSE multi-echo dataset with corresponding T2 maps.

Mean global T2 relaxation time (T2-RT) over all volunteers was 53.3 ± 2.7 ms (for the 9 Ec variant), 52.2 ± 2.0 ms (6 Ec), 51.3 ± 3.3 ms (6 Ec LR), and 50.4 ± 2.7 ms (7 Ec no s.e.) with the lowest inter-subject variation in the 6 Ec variant. T2 relaxation times for the 9 Ec sequence were significantly longer compared to all other variants. No significant differences were found between the two variants with six echoes. Bland-Altman analysis of the inter-study repeatability revealed a mean difference and 95% limits of agreement (LoA) between the first and the second examination of −0.04 ms [−8.4; 8.3] ms for the 9 Ec variant, −0.02 ms [−4.7; 4.7] ms for the 6 Ec variant, 1.0 ms [−5.9; 7.9] ms for the 6 Ec LR variant, and 2.2 ms [−1.0; 5.5] ms for the 7 Ec no s.e. variant. Considering the results of the phantom study and the T2 analysis in healthy volunteers including the assessment of inter-study repeatability, the six-echo (6Ec) variant was selected for further analysis.

**Figure 3:**
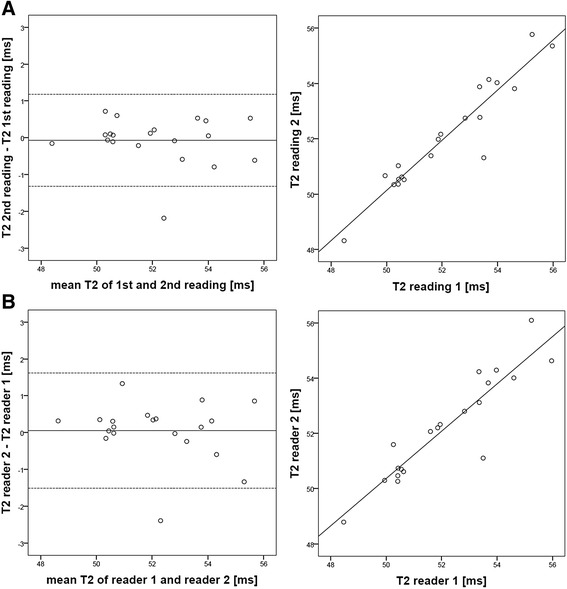
**Assessment of intra- and inter-reader agreement for global myocardial T2 measurements with the six-echo GraSE sequence. A**: Bland-Altman analysis shows very good agreement of repeated observations for the same reader (mean difference = −0.1 ms, 95% LoA = [−1.3; 1.2] ms) with an excellent linear correlation (R = 0.95). **B**: The agreement of two different readers is only slightly lower (R = 0.92) than repeated readings by the same reader.

The assessment of intra- and inter-observer agreement for the six-echo GraSE sequence revealed very small differences between repeated readings of the same reader (−0.1 ms, 95% LoA = [−1.3; 1.2] ms) and between the measurements of different readers (0.1 ms, 95% LoA = [−1.5; 1.6] ms), with a linear correlation coefficient of R = 0.95 and R = 0.92, respectively. The Bland-Altman plots and the corresponding correlation plots are displayed in Figure 3.

**Figure 4:**
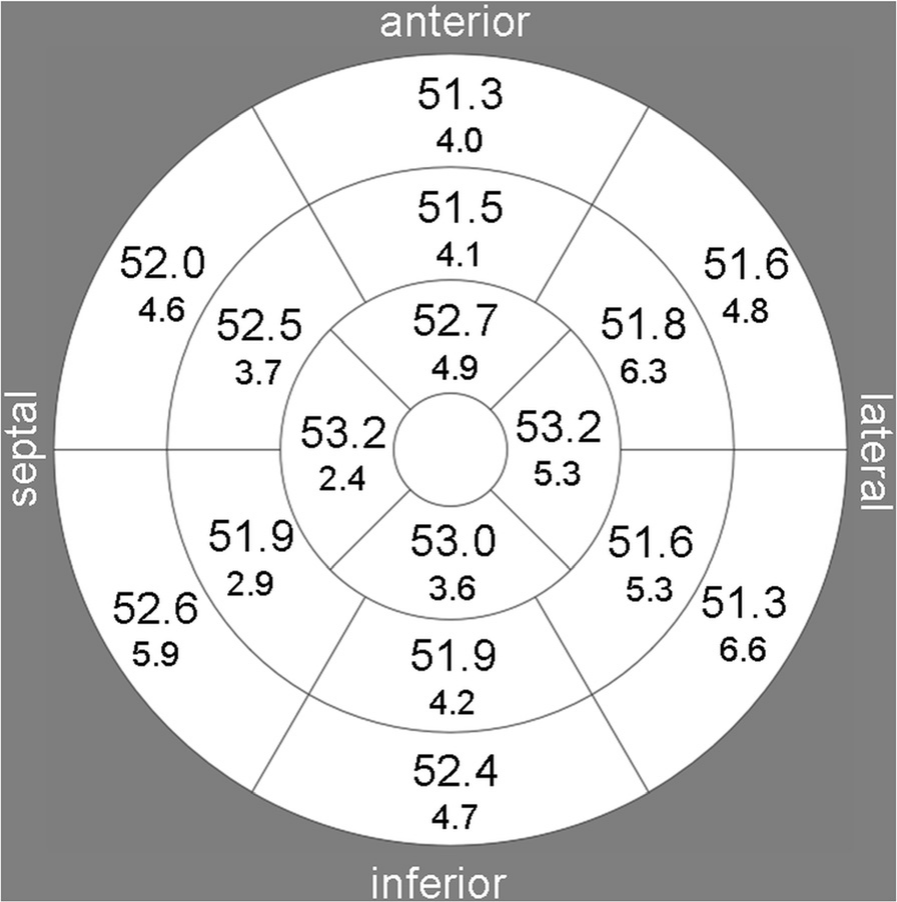
**Segmental analysis of myocardial T2 relaxation time.** Regional dependence of measured T2 values is illustrated using the 16 segment AHA model [20]. Slightly higher values for the T2 relaxation time of myocardium were observed in the apical segments, but with no significant difference to mid-ventricular and basal segments.

T2 values for the individual segments are presented in Figure 4. For segmental measurements the mean differences in T2-RTs between repeated readings were 0.04 ms, 95% LoA = [−2.8; 2.9] ms (same reader) and −0.1 ms, 95%LoA = [−4.4; 4.6] (different readers). Analysis of the regional variation in myocardial T2 relaxation time revealed a rather homogeneous distribution. Mean T2 was slightly higher in apical slices compared to mid ventricular slices (∆T2 = 1.2 ± 2.7 ms) and also compared to basal slices (∆T2 = 1.2 ± 4.9 ms) but the differences did not reach statistical significance. Differences between septal and lateral segments and apical and inferior segments were less than 1 ms.

### Clinical application

**Figure 5:**
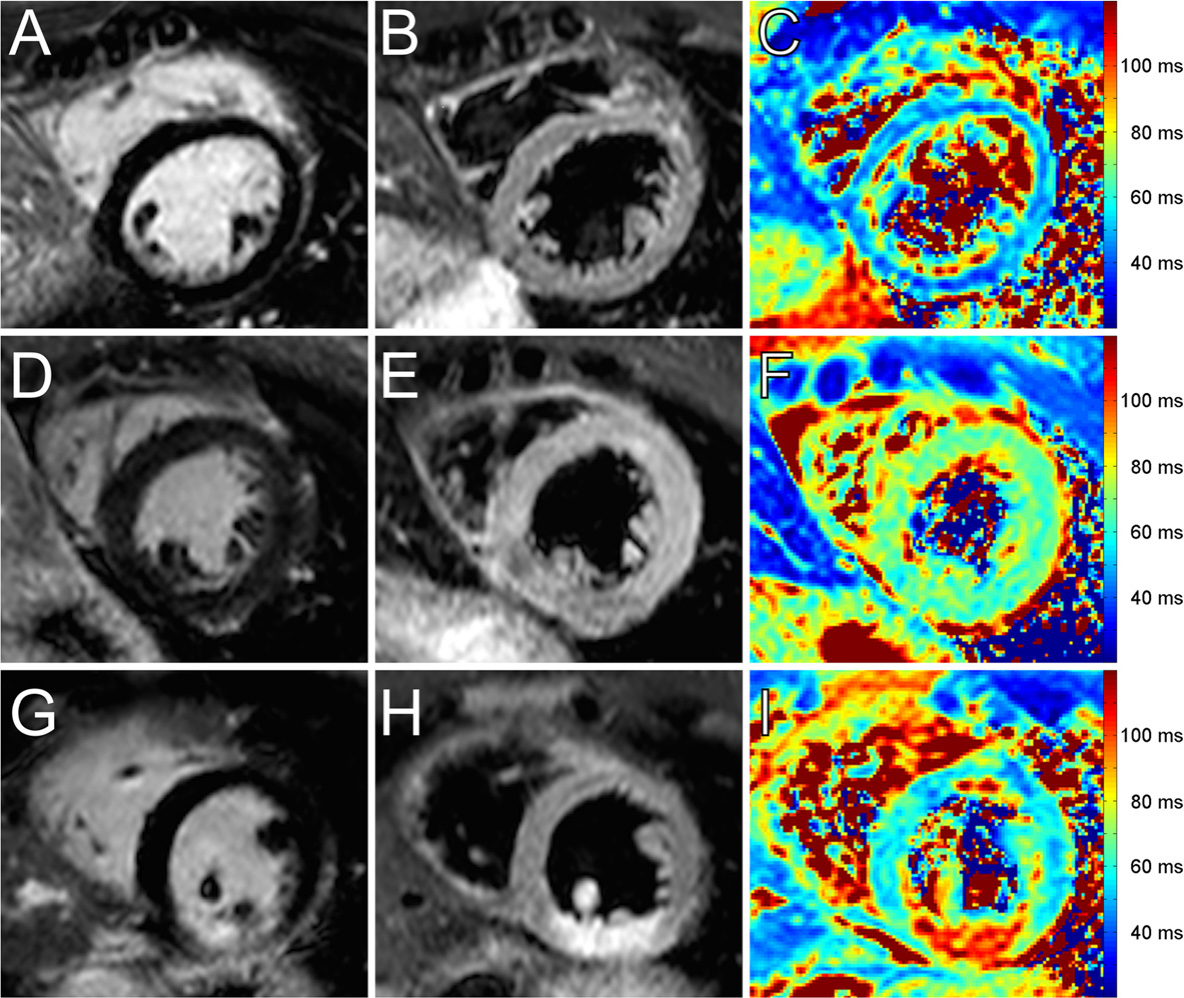
**Example CMR findings.** Midventricular short axis T2-weighted short tau inversion recovery (STIR), late gadolinium enhancement (LGE), and T2 mapping sequences in a 29-year-old healthy male volunteer **(A, B, C)**, a 42-year-old patient (#1) with acute, diffuse myocarditis **(D, E, F)**, and a 53-year-old patient (#2) with acute myocardial infarction **(G, H, I)**. Mean global T2 relaxation time for the healthy volunteer was 48.5 ± 4.6 ms (anterior: 47.8 ms; anteroseptal: 57.8 ms; inferoseptal: 52.1 ms; inferior: 49.6 ms; inferolateral: 45.5 ms; anterolateral: 47.1 ms) **(C)**. In patient #1 diffuse myocardial edema was present throughout all myocardial segments **(D, E, F)**, associated with increased global T2 values of 68.0 ± 2.4 ms (anterior: 65.4 ms; anteroseptal: 68.4 ms; inferoseptal: 70.5 ms; inferior: 65.8 ms; inferolateral: 64.4 ms; anterolateral: 66.1 ms). Patient #2 showed an acute myocardial infarction of the inferior segments with subendocardial LGE **(G)** and pronounced edema in the T2 STIR image **(H)**. Pronounced myocardial edema is also visible in the corresponding T2 map **(I)**, where the infarcted segments showed mean T2 relaxation times of 89.4 ms (inferior) and 82.3 ms (inferolateral) (anterior: 55.5 ms; anteroseptal: 56.2 ms; inferoseptal: 70.5 ms; anterolateral: 66.1 ms; global T2 relaxation time: 62.5 ± 13.1 ms).

After implementing the six-echo variant into clinical routine protocol, myocardial T2 mapping was performed in five patients with acute myocardial injury. Mean time from onset of symptoms to CMR was 2.2 ± 0.7 days. Global myocardial T2 relaxation times were considerably prolonged in patients with acute myocardial conditions (myocarditis: 62.9 ± 5.5 ms; myocardial infarction: 58.7 ± 8.4 ms; takotsubo cardiomyopathy: 63.5 ± 6.0 ms) (see Figure 5). In addition, T2 relaxation times were significantly prolonged in infarcted segments compared to remote segments (76.2 ± 11.1 ms vs. 53.4 ± 4.1 ms, p < 0.001). More detailed patient characteristics are given in Table 2.

**Table 2:** **Patient characteristics**

**Patient**	**Diagnosis**	**Gender**	**Age**	**ECG changes**	**Troponin I [ng/ml]**	**CRP [mg/l]**	**T2 total [ms]**
#1	Acute, diffuse myocarditis	Female	42	None	1.6	188.0	68.0 ± 2.4
#2	Acute myocardial infarction	Female	53	ST segment elevation	30.1	3.2	62.5 ± 13.1
#3	Takotsubo cardiomyopathy	Female	44	Left bundle branch block	9.6	5.1	63.5 ± 6.0
#4	Acute myocarditis	Male	28	None	4.1	59.7	57.7 ± 8.7
#5	Acute myocardial infarction	Male	68	T-wave abnormalities	5.0	4.1	54.8 ± 4.1

Patient characteristics for five patients with acute myocardial injury. Myocardial T2 relaxation times were obtained with the six-echo variant of the GraSE sequence.

CRP = C-reactive protein; ECG = Electrocardiogram; T2 = Global myocardial T2 relaxation times.

### Image quality analysis

Image quality scores of the T2 maps showed no significant difference between healthy volunteers and patients with acute myocardial injuries (4.5 ± 0.6 vs. 4.4 ± 0.6). No significant differences were found between basal, midventricular, and apical sections (4.3 ± 0.8 vs. 4.6 ± 0.5 vs. 4.5 ± 0.5).

## Discussion

In this prospective study, the applicability of myocardial T2 mapping using the GraSE technique was systematically evaluated. The specific sequence parameters derived from sequence optimization allowed for an accurate estimation of myocardial T2 relaxation times in clinically acceptable breath-hold times. After a phantom pre-study and the evaluation of four different sets of sequence parameters in an in-vivo volunteer study, the six-echo variant of the GraSE sequence was judged to offer the best performance for clinical use when taking into account the measurement accuracy and repeatability. The use of the six-echo variant was demonstrated in five clinical cases.

The nine-echo variant suffered from a relatively high inter-study variance. This may be attributed to the relatively long shot duration of 118 ms making it more susceptible to motion artifacts. Subsequently, this increased susceptibility to motion artifacts may counterbalance if not even outweigh the benefit of acquiring more data points for estimation of T2. The evaluation of the six-echo variant with lower resolution revealed no benefit with respect to inter-study reproducibility and inter-subject variability. Here, the gain in SNR appears to be counteracted by partial-volume effects. The omission of a start-up echo yielded only a slight overestimation of T2-RT in the phantom measurements, therefore we also evaluated a variant without start-up echo in the in-vivo experiments, again with the aim to increase the SNR. Interestingly, the omission of a start-up echo did not lead to higher values for T2 in the in-vivo experiments, but also did not show a distinct benefit.

In existing literature to date a large range of myocardial T2 values for healthy volunteers is reported. At a field-strength of 1.5 Tesla, T2 values from 50 ms [7] to 59 ms [22] are reported. However, comparably long T2 values of 59 ms are elsewise only reported for lower field-strengths [23], suggesting that a T2 relaxation time of 59 ms may rather be regarded as an outlier. Three possible explanations exist: (1) In the phantom measurements performed for sequence optimizaton we could demonstrate that the combination of a short first TE and a suboptimal slice profile leads to a severe overestimation of T2-RTs. Because a first TE of 7 ms was chosen [22], the high T2-RT may be caused by a suboptimal slice profile of the navigator-gated GraSE sequence. (2) T2 was estimated by exponential fitting without accounting for the non-Gaussian noise distribution in magnitude images [18]. (3) Two studies reported a correlation of myocardial T2 with age [22,23] indicating that normal T2-RTs may be age-dependant. However, this was not confirmed in a different study [24], leaving the impact of age on T2-RTs unclear at this time. The shortest T2 values of 50 ms are reported in a study where a steady state free precession (SSFP) sequence was applied with interleaved variation of inversion time and T2 preparation time for simultaneous mapping of T1 and T2 relaxation times [7]. Interestingly, Giri et al. reported values for T2 in healthy volunteers very similar to our results [10], although the parameters of the SSFP sequence used for in-vivo T2 relaxation time mapping in that study showed only inferior accuracy in phantom measurements. In a very recent study a nine-echo navigator-gated sequence was used successfully to detect myocardial edema [25]. Although the healthy control group was very similar to the volunteers examined in our study with respect to age and sex, T2 values observed for healthy myocardium were distinctly longer (interquartile range: [54; 60] ms) compared to our measurements (interquartile range: [50.4; 53.6] ms). As the echo spacing was only 6.25 ms in that study, the longer values may again be an effect of a suboptimal slice profile. Unfortunately, there was no phantom validation performed in that study [26].

The segmental analyses reported in our study revealed only minor regional variations in healthy volunteers with slightly higher T2 relaxation times in apical segments compared to mid-ventricular and basal segments, similar to previous studies [10,22], including a 3 Tesla study [24]. Knobelsdorff et al. speculated that the regional variation is most probably caused by partial-volume effects increasing towards the apex [24]. A tendency to a higher inter-subject variation in lateral segments is most probably an effect of wall motion, and was also observed in SSFP-based T2 mapping [10].

T2 mapping based on the GraSE sequence has several inherent pitfalls that must be addressed. First, the use of an EPI readout introduces some T2* weighting and can lead to an underestimation of T2 relaxation times. However, clinically acceptable breath-hold durations can already be achieved with a relatively modest EPI factor of 7, as applied in this study. In contrast, the use of a turbo-spin echo series for T2 mapping can result in a prolongation of observed T2 relaxation time, depending on echo spacing and the applied turbo factor [27]. The GraSE sequence variant used in our study did not show a systematic deviation from the reference T2 determined in the phantom experiment. However, a less homogenous B_0_ field in in-vivo applications may introduce a slight underestimation of T2. Another drawback of the GraSE sequence is that each echo is acquired at a slightly different heart phase. However, with the six-echo variant, the shot duration is less than 85 ms, which should not introduce a significant error in T2 estimation, as long as the trigger delay is set appropriately to achieve image acquisition in the end diastole.

One problem using qualitative T2-w imaging is that subendocardial edema may be difficult to differentiate from low flow left ventricular blood [5], possibly also making contouring of the myocardial borders error-prone. However, the high intra- and inter-reader agreements demonstrated for the six-echo variant indicate that reliable contouring of the subepicardial and subendocardial borders of the left ventricle can be performed even by investigators with different levels of experience. Moreover, while correct interpretation of standard qualitative T2-w images is challenging in global edema due to the lack of healthy reference tissue, we demonstrated that diffuse myocardial edema as seen in patient #2 can be detected more easily with myocardial T2 mapping compared to qualitative T2-w imaging. Although some clinical studies already investigated the diagnostic value of myocardial T2 mapping in acute myocardial injury [11,25,28] more studies with well-defined study populations will be necessary to evaluate the clinic impact of myocardial T2 mapping [26]. Especially studies, with well-defined cohorts that use a longitudinal design will be necessary to investigate the benefits for monitoring therapy.

Our study has several limitations. First, the reference values provided in this study are mainly for younger male adults. The reference values can probably not be generalized to older and female patients without further validation. Second, it should be noted that arrhythmia may degrade accuracy and robustness of T2 measurements.

## Conclusion

In conclusion, our study shows that myocardial T2 mapping using the GraSE technique can be performed within an acceptable acquisition time. However, appropriate sequence settings are necessary to obtain accurate and reliable results. The technique demonstrated here has the potential to overcome some limitations of qualitative T2-w imaging, and may further enhance the diagnostic quality of CMR in patients with suspected acute myocardial injuries.

## Additional file

Additional file 1:
**Illustration of the GraSE sequence and phantom study details.**

## Abbreviations

GraSE: Gradient-spin-echo
TSE: Turbo-spin-echo
CMR: Cardiovascular magnetic resonance
EPI: Echo-planar-imaging
TE: Echo time
T2-RT: T2 relaxation time
SPIR: Spectral selective inversion recovery
STIR: Short tau inversion recovery
SSFP: Steady state free precession
LoA: Limits of agreement
9Ec: Nine echo variant
6Ec: Six echo variant
6Ec LR: Six echo variant with lower resolution
7Ec no s.e.: Seven echo variant without start-up echo

## References

[1] Ferreira VM, Piechnik SK, Dall’Armellina E, Karamitsos TD, Francis JM, Choudhury RP (2012). Non-contrast T1-mapping detects acute myocardial edema with high diagnostic accuracy: a comparison to T2-weighted cardiovascular magnetic resonance. J Cardiovasc Magn Reson.

[2] Luetkens JA, Doerner J, Thomas DK, Dabir D, Gieseke J, Sprinkart AM et al. Acute myocarditis: multiparametric cardiac MR imaging. Radiology. 2014;273(2):383-392. 10.1148/radiol.14132540 24910904

[3] Higgins CB, Herfkens R, Lipton J, Sievers R, Sheldon P, Kaufman L (1983). Nuclear magnetic resonance imaging of acute myocardial infarction in dogs: alterations in magnetic relaxation times. Am J Cardiol.

[4] Eitel I, Friedrich MG (2011). T2-weighted cardiovascular magnetic resonance in acute cardiac disease. J Cardiovasc Magn Reson.

[5] Abdel-Aty H, Simonetti O, Friedrich MG (2007). T2-weighted cardiovascular magnetic resonance imaging. J Magn Reson Imaging.

[6] Huang TY, Liu YJ, Stemmer A, Poncelet BP (2007). T2 measurement of the human myocardium using a T2-prepared transient-state TrueFISP sequence. Magn Reson Med.

[7] Blume U, Lockie T, Stehning C, Sinclair S, Uribe S, Razavi R (2009). Interleaved T-1 and T-2 relaxation time mapping for cardiac applications. J Magn Reson Imaging.

[8] Feng L, Otazo R, Jung H, Jensen JH, Ye JC, Sodickson DK (2011). Accelerated cardiac T2 mapping using breath-hold multiecho fast spin-echo pulse sequence with k-t FOCUSS. Magn Reson Med.

[9] He T, Gatehouse PD, Anderson LJ, Tanner M, Keegan J, Pennell DJ (2006). Development of a novel optimized breathhold technique for myocardial T2 measurement in thalassemia. J Magn Reson Imaging.

[10] Giri S, Chung YC, Merchant A, Mihai G, Rajagopalan S, Raman SV, et al. T2 quantification for improved detection of myocardial edema. J Cardiovasc Magn Reson. 2009;11:56. 10.1186/1532-429X-11-56 PMC2809052 20042111

[11] Thavendiranathan P, Walls M, Giri S, Verhaert D, Rajagopalan S, Moore S (2012). Improved detection of myocardial involvement in acute inflammatory cardiomyopathies using T2 mapping. Circ Cardiovasc Imaging.

[12] Van Heeswijk RB, Feliciano H, Bonanno G, Coppo S, Lauriers N, Locca D (2009). Quantitative free-breathing 3 T T2-mapping of the heart designed for longitudinal studies. J Cardiovasc Magn Reson.

[13] Verhaert D, Thavendiranathan P, Giri S, Mihai G, Rajagopalan S, Simonetti OP (2011). Direct T2 quantification of myocardial Edema in acute ischemic injury. JACC Cardiovasc Imaging.

[14] Feinberg DA, Oshio K (1991). GRASE (gradient-and spin-echo) MR imaging: a new fast clinical imaging technique. Radiology.

[15] Oshio K, Feinberg DA (1991). GRASE (Gradient-and Spin-Echo) imaging: a novel fast MRI technique. Magn Reson Med.

[16] Hennig J, Nauerth A, Friedburg H (1986). RARE imaging: a fast imaging method for clinical MR. Magn Reson Med.

[17] Mansfield P (1977). Multi-planar image-formation using Nmr Spin echoes. J Phys C Solid State Phys.

[18] Bos C, Duijndam A, Sénégas J (2009). Reference phantom validation of T2-mapping: maximum likelihood estimation of T2 from magnitude phased-array multi-echo data. Proceedings of the 17th Annual Meeting of ISMRM, Honolulu.

[19] Hardy PA, Andersen AH (2009). Calculating T2 in images from a phased array receiver. Magn Reson Med.

[20] Cerqueira MD, Weissman NJ, Dilsizian V, Jacobs AK, Kaul S, Laskey WK (2002). Standardized myocardial segmentation and nomenclature for tomographic imaging of the heart a statement for healthcare professionals from the cardiac imaging committee of the Council on Clinical Cardiology of the American Heart Association. Circulation.

[21] Heiberg E, Sjögren J, Ugander M, Carlsson M, Engblom H, Arheden H (2010). Design and validation of Segment-freely available software for cardiovascular image analysis. BMC Med Imaging.

[22] Bönner F, Neizel M, Gruenig S, Jacoby C, Kelm M, Sievers G (2013). T2 mapping in different cardiomyopathies: first clinical experience. J Cardiovasc Magn Reson.

[23] Mavrogeni S, Tzelepis GE, Athanasopoulos G, Maounis T, Douskou M, Papavasiliou A (2005). Cardiac and sternocleidomastoid muscle involvement in Duchenne muscular dystrophy - An MRI study. Chest.

[24] von Knobelsdorff-Brenkenhoff F, Prothmann M, Dieringer MA, Wassmuth R, Greiser A, Schwenke C (2013). Myocardial T1 and T2 mapping at 3 T: reference values, influencing factors and implications. J Cardiovasc Magn Reson.

[25] Radunski UK, Lund GK, Stehning C, Schnackenburg B, Bohnen S, Adam G (2014). CMR in patients with severe myocarditis: diagnostic value of quantitative tissue markers including extracellular volume imaging. JACC Cardiovasc Imaging.

[26] Raman SV, Siddiqui Y (2014). Mapping myocarditis: still searching for the North Star*. JACC Cardiovasc Imaging.

[27] Träber F, Block W, Layer G, Bräucker G, Gieseke J, Kretzer S (1996). Determination of 1H relaxation times of water in human bone marrow by fat-suppressed turbo spin echo in comparison to MR spectroscopic methods. J Magn Reson Imaging.

[28] Naßenstein K, Nensa F, Schlosser T, Bruder O, Umutlu L, Lauenstein T (2014). Cardiac MRI: T2-mapping versus T2-weighted dark-blood TSE imaging for myocardial edema visualization in acute myocardial infarction. Fortschr Röntgenstr.

