# Supplementary material for: Gradient Spin Echo (GraSE) imaging for fast myocardial T2 mapping
Source: J Cardiovasc Magn Reson. 2015 Feb 12;17(1):12. doi: 10.1186/s12968-015-0127-z (PMC4326516; doi:10.1186/s12968-015-0127-z)
Supplement: Additional file 1: — Illustration of the GraSE sequence and phantom study details. [file 12968_2015_127_MOESM1_ESM.doc]

**Additional file 1** Illustration of the GraSE sequence and phantom study details

**GraSE sequence for myocardial T2 mapping**

An illustration of the sequence scheme applied in this study is shown in Figure A1.

**
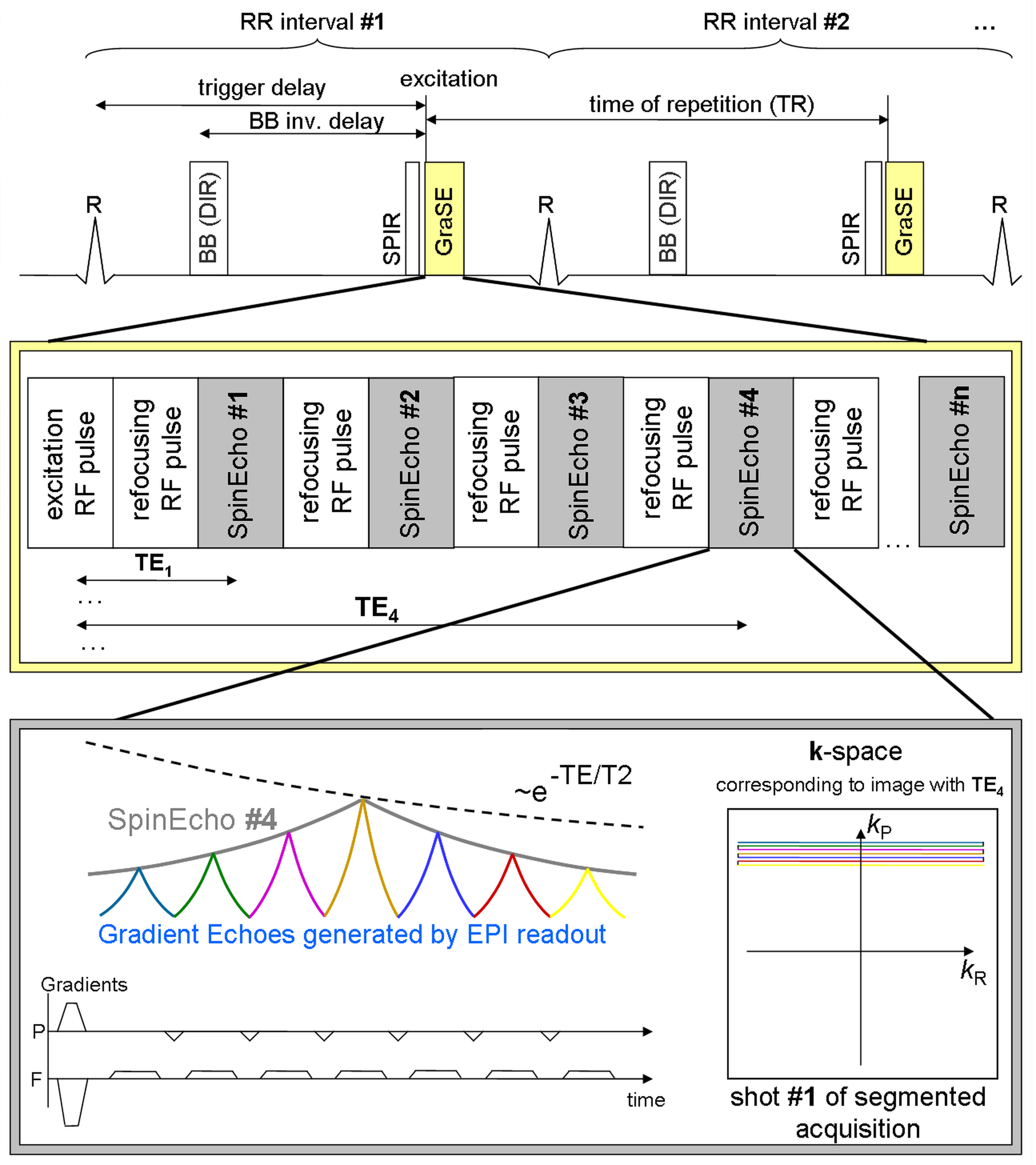
**

**Figure A1. Illustration of Gradient Spin Echo Imaging for Myocardial T2 Mapping.** A train of spin-echoes is generated in each RR interval (yellow box) where the number of spin-echoes is determined by the turbo-spin-echo (TSE-) factor. Note that the TSE-factor equals the number of images in the multi-echo series. The trigger delay should be optimized by analysis of cine MRI data to generate this train of spin echoes in a cardiac phase were only minimal motion is present. Each of the spin-echoes is sampled by an echo-planar imaging (EPI) readout module (gray box). The EPI-module generates a number (=EPI-factor) of gradient-echoes. Since the EPI-factor is lower than the number of lines in k-space, this procedure has to be repeated until all profiles are acquired (multi-shot acquisition), e.g. with a matrix size of 168 in phase encoding direction, a parallel imaging factor of 2, and an EPI factor of 7, the acquisition time amounts to 168/2/7 = 12 RR intervals plus 1 dummy TR (not shown in the illustration). For clarity of illustration, start-up echoes are also omitted in the illustration. If the number of start-up echoes equals 1, data at TE1 are discarded from analyses. BB (DIR): Black Blood (Dual Inversion Recovery), SPIR: Spectral Presaturation with Inversion Recovery for fat suppression.

**Phantom pre-study**

A Carr-Purcell-Meiboom-Gill sequence with 8 echoes (TE_min_/∆TE = 15 ms/15 ms, TR = 2000 ms) served as reference for all phantom measurements. Preliminary phantom experiments were performed to determine a suitable set of sequence parameters for accurate T2 mapping with GraSE. To obtain an optimal signal-to-noise ratio, we first sought to set the echo time of the first echo and the echo spacing to the minimum value achievable by full gradient power and default RF-pulse mode. However, in this setting very short RF pulses are applied, leading to a slice profile insufficient for accurate T2 mapping. An imperfect slice profile causes an inhomogeneous flip angle distribution in through-plane direction which can lead to severe signal alterations. At the employed MR system, adequate RF pulses can be achieved by either setting the parameter "slice-profile" to "optimal profile" (a parameter which is visible for the user in an advanced parameter setting mode) or by setting the minimum echo time to a higher value (here: 11.8 ms), which automatically leads to the application of RF pulses that yield a better slice profile.

Of note, if no dummy echo is applied (no startup echo) and the echo time is set to "minimum" without forcing the application of adequate RF pulses, the T2 relaxation time is severely overestimated (see Figure A2). Therefore, we strongly recommend users to check how to ensure that appropriate RF pulses are applied at their particular system to ensure a sufficient quality of the slice profile mandatory for accurate estimation of T2.


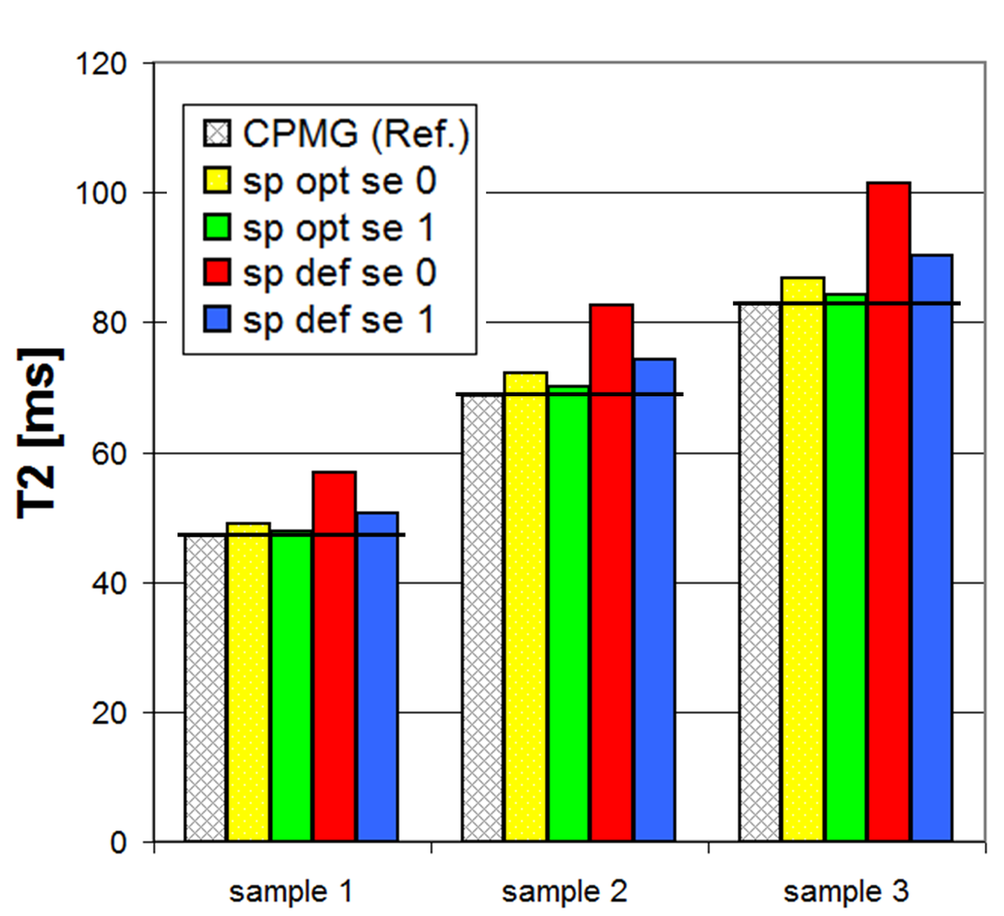


**Figure A2.** **Influence of slice profile setting and start-up echo on T2 measurement in a pre-study with three phantom tubes.** The horizontal black line represents the reference value for T2 for each of the three samples. When the echo time is set to minimum and no start up echo is applied, T2 is severely overestimated if the parameter "slice profile" is set to "default" (sp def se 0). T2 relaxation time is also overestimated if the slice profile parameter is set to "optimal", but no dummy echo is applied (sp opt se 0). The reason for this is that the first echo typically suffers from a signal attenuation, which leads to an overestimation of the T2 relaxation time.

| Myocardial Segment | 9 Ec | 6 Ec | 6 Ec LR | 7 Ec no s.e. |
| --- | --- | --- | --- | --- |
| basal anterior | 52.2 ± 4.3 | 51.3 ± 4.0 | 52.5 ± 7.1 | 50.8 ± 4.7 |
| basal anteroseptal | 53.2 ± 4.7 | 52.0 ± 4.6 | 53.1 ± 7.7 | 50.0 ± 3.8 |
| basal inferoseptal | 53.4 ± 3.4 | 52.6 ± 5.9 | 51.2 ± 3.2 | 51.5 ± 3.9 |
| basal inferior | 55.0 ± 4.1 | 52.4 ± 4.7 | 52.2 ± 4.2 | 51.1 ± 4.6 |
| basal inferolateral | 53.1 ± 5.6 | 51.3 ± 6.6 | 51.9 ± 7.6 | 50.0 ± 4.4 |
| basal anterolateral | 51.3 ± 5.3 | 51.6 ± 4.8 | 49.6 ± 4.8 | 48.4 ± 6.3 |
| mid anterior | 52.8 ± 2.8 | 51.5 ± 4.1 | 49.6 ± 5.4 | 49.9 ± 4.3 |
| mid anteroseptal | 51.8 ± 4.2 | 51.8 ± 6.3 | 49.3 ± 5.6 | 47.8 ± 5.7 |
| mid inferoseptal | 53.8 ± 3.2 | 51.9 ± 2.9 | 51.6 ± 2.7 | 51.7 ± 2.4 |
| mid inferior | 53.4 ± 2.3 | 51.9 ± 4.2 | 50.4 ± 4.0 | 51.2 ± 3.5 |
| mid inferolateral | 51.1 ± 4.0 | 51.6 ± 5.3 | 48.9 ± 5.3 | 47.8 ± 4.5 |
| mid anterolateral | 51.8 ± 4.2 | 51.8 ± 6.3 | 49.3 ± 5.6 | 47.8 ± 5.7 |
| apical anterior | 54.5 ± 3.7 | 52.7 ± 4.9 | 52.4 ± 4.7 | 51.1 ± 4.4 |
| apical septal | 55.1 ± 3.7 | 53.2 ± 2.4 | 52.4 ± 3.6 | 53.7 ± 3.4 |
| apical inferior | 54.5 ± 3.8 | 53.0 ± 3.6 | 52.7 ± 5.0 | 51.1 ± 3.2 |
| apical lateral | 54.5 ± 3.5 | 53.2 ± 5.3 | 51.7 ± 4.9 | 49.0 ± 4.7 |
| total | 53.3 ± 2.7 | 52.2 ± 2.0 | 51.3 ± 3.3 | 50.4 ± 2.7 |

**Table A1. Results of the segmental analyses for the four sequence variants evaluated within the in-vivo study.** T2 values are given in ms (mean ± standard deviation).
